# The Benefits of Prebiotics and Probiotics on Mental Health

**DOI:** 10.7759/cureus.43217

**Published:** 2023-08-09

**Authors:** Karlyle G Bistas, Jean Paul Tabet

**Affiliations:** 1 Psychiatry and Behavioral Sciences, Wayne State University Detroit Medical Center, Detroit, USA; 2 School of Medicine, Medical University of the Americas, Charlestown, KNA

**Keywords:** gut microflora, depression, anxiety, probiotics, prebiotics

## Abstract

In individuals with depression and anxiety, the composition or alteration of their gut microbiota can significantly affect their psychological symptoms. Articles for this study were selected using PubMed and NCBI (National Center for Biotechnology Information) with the following search terms: “gut microbiota,” “depression,” “anxiety,” “probiotics,” and “prebiotics.” These studies evaluated the composition of the gut microbiota and the decrease in symptoms of depression and anxiety due to treatment with probiotics and prebiotics. Only papers published after 2015 were included. There was a significant relationship between the composition and alteration of the gut microbiota and the presence or variation of symptoms of depression and anxiety. Treatment with probiotics or prebiotics improved the symptoms of these mental health conditions. This literature review examines how different prebiotics and probiotics affect mental health and how altering individuals’ gut microbiota correlates with depression and anxiety. Treatment with probiotics or prebiotics may decrease the severity of these mental disorders by altering the gut microbiota.

## Introduction and background

Depression and anxiety are the most prevalent mental health disorders among adults, with 5% of adults suffering from depression globally. These disorders are some of the leading causes of disability around the world [1]. Depression is a mental illness characterized by a persistently low mood. Typical symptoms include anhedonia, apathy, depressed mood, and psychomotor slowing. If left untreated, depression can have detrimental effects on numerous aspects of an individual’s life and interpersonal relationships. Depression is a multifactorial mental illness resulting from brain chemical abnormalities, genetics, psychosocial stressors, trauma, medical conditions, and certain medications [2]. Anxiety disorders are also one of the most common mental health disorders, affecting 19.1% of the US population of adults aged 18 years or older [2]. Anxiety is described as a state of intense fear. When it becomes excessive and interferes with daily functioning, it becomes a disorder. The total global economic cost from the loss of productivity that results from anxiety and depression amounts to US$1 trillion each year [3]. Standard treatment for depression and anxiety includes drugs that alter the neurotransmitter levels in the brain, including increasing serotonin, norepinephrine, and dopamine, as well as other non-pharmacological treatments like cognitive behavioral therapy. Antidepressants and anxiolytics are known to be more effective than placebos. Some of the side effects of these medications include excessive nausea, weight gain, drowsiness, insomnia, dry mouth, constipation, dizziness, agitation, restlessness, and sexual side effects [4].

Recent literature has shown the importance of the relationship between gut microbiota and mental illnesses [5]. The brain-gut axis communicates in a bidirectional manner. This link is further established by studies showing there is a high rate of co-morbidity among individuals with anxiety and depression and gastrointestinal diseases such as irritable bowel syndrome and inflammatory bowel disease [6]. Microbiota are microorganisms, including bacteria and archaea, that live in the digestive tract of humans. These microorganisms have been the topic of numerous studies due to their significant connection to human physiology. Although current knowledge about gut microbiota is still under review, an increasing number of studies show the relationship between gut microbiota and physical and mental health. The composition of the microbiota is influenced by numerous factors, including genetics, age, sex, diet, and stress levels [7]. The gastrointestinal tract has a large surface area and is exposed to commensal microorganisms as well as exogenous pathogens. The number of bacteria in the gut is greater than the actual number of somatic cells in the human body [8]. These bacteria have diverse functions, such as regulating host nutrient metabolism, maintaining the mucosal barrier, immunomodulation, and defending against pathogenic organisms [9]. Probiotics are defined as live microorganisms with beneficial health effects on the host, and prebiotics are given to stimulate the growth of healthy bacteria in the gut. The gut microbiota is defined as microorganisms that live in the gut.

Studies have shown that depression can increase the permeability of the gut barrier, resulting in bacteria seeping into the circulation and producing an inflammatory response through an immune response and endotoxin translocation [10]. Anxiety also increases gut permeability, resulting in the same inflammatory response [11]. A study found that stress-exposed mice had increased circulating levels of the cytokine interleukin-6. This interleukin plays a central role in host defense. Furthermore, studies have shown that higher levels of interleukin-6 and C-reactive protein led to the development of depressive symptoms [12]. Moreover, depression, as an independent factor, causes higher levels of inflammatory cytokines in individuals. Stress can lead to a larger inflammatory response, which has also been linked to anxiety and depression.

Furthermore, cytokines induce depressive and anxiety symptoms by altering the activity of neurotransmitters. For example, cytokines stimulate indoleamine 2,3-dioxygenase, which decreases serotonin production by increasing the tryptophan metabolite kynurenine [13]. Increased availability of tryptophan leads to increased serotonin availability, which can reduce depressive symptoms. In addition to increasing gut permeability and causing inflammation, studies have shown that stress-induced permeability allows exogenous pathogens to reach the central nervous system (CNS). This causes an increased sensitivity in the hypothalamic-pituitary-adrenal axis, which may augment levels of corticotrophin-releasing factor [14]. Corticotrophin-releasing hormone and cortisol were both found to increase intestinal permeability in humans [15].

Furthermore, the gut microbiota plays a role in disorders related to the brain. Dysregulation in immune regulation, signaling pathways, and gastrointestinal function may occur due to a disturbance of the gut microbiota. Modulating the gut microbiota in patients with depression or anxiety may prove useful, as evidenced by the significant relationship between the gut and the CNS. Several studies of patients with depression and anxiety have shown the positive effect of probiotics on modulating the symptoms of these disorders [16]. This paper is a literature review and analyzes the effect of the composition and alteration of the gut microbiota on the symptoms of depression and anxiety.

## Review

Methods

This paper is a literature review and reviews articles based on the inclusion and exclusion criteria discussed below. The articles for this paper were chosen based on whether they evaluated the composition of gut microbiota and their effects on symptoms of anxiety and depression. The keywords used in the search queries included “gut microbiota,” “depression,” “anxiety,” “probiotics,” and “prebiotics,” and related articles were identified by searching PubMed, PubMed Central, NCBI (National Center for Biotechnology Information), and Google Scholar. Boolean terms included “And, Or, Not.” Double- and triple-blind studies were used, and methods for measuring depression and anxiety were specified according to each paper. The study population consisted of adults aged 18 years and older, and inclusion criteria included studies that were peer-reviewed, written in English, and published after 2015. Exclusion criteria included studies that were published in 2015 or before. Based on these criteria, a search of the databases yielded nine clinical trials and one case-control study, which were used in this study. Two specific inventories were utilized to quantify depression and anxiety. The Beck’s Depression Inventory (BDI) is a questionnaire used to analyze depression and the Hamilton Depression Rating Scale (HDRS) was used to determine if symptoms of anxiety are present and what the severity of the symptoms are. The results were organized into an evidence table and reviewed nine randomized clinical trials and one case-control study.

Results

A double-blind, randomized clinical trial was completed to determine the impact of probiotic and prebiotic supplements on Beck’s score [17]. The total BDI score was added together for each item, where 0-9 individuals indicated minimal depression, 10-18 individuals indicated mild depression, 19-20 individuals indicated moderate depression, and 30-63 individuals indicated severe depression [18]. The study observed 110 depressed patients who were randomly assigned to receive *Lactobacillus helveticus* and *Bifidobacterium longum* probiotics (n = 38) with a dosing of 10 x 109, galactooligosaccharide prebiotics (n = 36), or a placebo (n = 36) with xylitol, maltodextrin, plum flavor, and malic acid for eight weeks [18]. All treatments were taken daily. Of the 110 patients included in the study, 81 completed the trial [18]. The patients’ ages ranged from 18-50 years old, with an average age of 37 years. All patients had mild to moderate major depression and took antidepressant drugs (sertraline, fluoxetine, citalopram, or amitriptyline) for a minimum of three months before starting the trial. Exclusion criteria included major organ disease, alcohol or tobacco intake, or inflammatory diseases.

The results concluded that there was a significant decrease in the overall BDI score of individuals on probiotic supplements (baseline: 18.25; end: 9.0) (p = 0.05), with no significant effect of the prebiotic supplementation or placebo [18]. This suggests that the probiotic significantly improves symptoms of depression. A secondary outcome measured participants’ serum kynurenine-to-tryptophan ratio [13]. A significant change was seen in the probiotic group, which demonstrated a decreased kynurenine-to-tryptophan ratio when adjusted for serum isoleucine [13]. This may indicate that tryptophan shunts the production of kynurenine, which leads to serotonin deficiency [13]. It appears that probiotics increase the amount of tryptophan that can be converted to serotonin by reducing the number of enzymes that convert tryptophan to kynurenine [13]. This implies that the improved depression scores may be due to the decrease in the kynurenine-to-tryptophan ratio.

A double-blind, randomized clinical trial was conducted to study the effect of symbiotic supplementation (combination of prebiotics and prebiotics) on fluoxetine in patients with moderate depression [19]. The prebiotic that was used contained *Lactobacillus casei* (3 x 108), *Lactobacillus acidophilus* (2 x 108), *Lactobacillus bulgaricus* (2 x 109), *Lactobacillus rhamnosus* (3 x 108), *Bifidobacterium breve* (2 x 108), *Bifidobacterium longum* (1 x 109), *Streptococcus thermophilus* (3 x 108), and 100 mg of fructooligosaccharide, while the placebo contained 1000 mg of magnesium stearate [19]. A total of 40 patients with moderate depression were recruited, with 20 patients in the treatment group and 20 in the placebo group. Exclusion criteria included patients who experienced schizophrenia, bipolar disorders, or cognitive disorders within the past year, along with pregnant and lactating women [19]. The treatment group was treated with the above prebiotics daily for six weeks, following four weeks of treatment with fluoxetine, and the outcome was measured using the HDRS [19]. A score of 10-13 indicated mild depression, 14-17 indicated mild to moderate depression, and >17 indicated moderate to severe depression [19]. At the end of the study, the symbiotic group experienced a clinically significant decrease in the Hamilton rating scale for depression (HAM-D) score compared to the placebo group (-19.25 in treatment versus -17.75 in placebo) (p = 0.024) [19]. Interestingly, while measurements for gender, age, BMI, and baseline HAM-D were taken, no baseline microbiota measurement was established before the study [19]. Of the individuals, 70% were female, and the mean age was 35 years, with no significant difference observed in the groups [19]. Side effects included nausea, bloating, diarrhea, and abdominal cramps, with the differences between the treatment and placebo not being significant [19]. The study showed a positive effect of symbiotics as an adjuvant therapy for moderate depression [19].

The efficacy of a four-week-long daily regimen of 5 g of short-chain fructooligosaccharide (scFOS) prebiotics on patients with irritable bowel syndrome (IBS) and anxiety was studied in a double-blind, randomized clinical trial [20]. The study measured anxiety using the Hospital Anxiety and Depression Scale, which is a questionnaire with seven items measuring depression with an anxiety subscale [20]. Scoring ranges from 0 to 3, with 3 indicating the highest level of anxiety or depression [20]. Subjects’ ages ranged from 18 to 60 years old. Patients’ fecal bacteria populations were studied using polymerase chain reaction (PCR) to test DNA from fecal extractions [20]. The bacteria tested included *Eubacteria*, bifidobacteria, *Lactobacilli*, *Enterobacteriaceae*, *Roseburia*, and *Faecalibacterium prausnitzii* [20]. This ensured a similar baseline of microbiota, which allowed for a better conclusion regarding the effect of the treatment. Exclusion criteria were subjects receiving treatment for depression, those with organic intestinal disease, and those with a history of alcohol and tobacco abuse [20]. The patients were required to follow a diet consisting of less than 20 grams of dietary fiber daily before the study period. They were also required to take a rectal hypersensitivity test using a premade screening test. The study was carried out on 41 subjects in the scFOS group and 38 subjects who received the placebo [20].

The results concluded that the scFOS group experienced a significantly improved quality of life and rectal comfort compared to the placebo group [20]. Moreover, the scFOS group experienced significantly reduced anxiety scores (approximately -2) and increased bifidobacteria compared to the placebo group, with -0.05 (p = 0.037) [20]. Interestingly, the study found an inverse correlation between the level of fecal bifidobacteria and abdominal pain and that patients with IBS experience reduced levels of the bacteria [20]. This implies that reduced levels of bifidobacteria may be correlated to higher levels of symptomatic anxiety and pain [20]. Subjects had normal depression scores according to the Hospital Anxiety and Depression Scale at the beginning of the study, which may be the reason for no change in depression scores [20].

A double-blind, randomized clinical trial was carried out to study the effect of a 10-week daily dose of a *Bifidobacterium longum* probiotic (1010 colony forming unit (CFU)/1 g power with maltodextrin) on patients with IBS, mild to moderate anxiety, or depression based on the Hospital Anxiety and Depression Scale [21]. Quality of life was also measured using self-questionnaires along with a functional magnetic resonance imaging test performed to observe brain activation patterns [21]. Serum cytokines and C-reactive protein (CRP) were also measured, along with human brain-derived neurotrophic factors [21]. Patients with organic diseases and those who used immunosuppressants, corticosteroids, antidepressants, anxiolytics, alcohol, or illegal drugs were excluded from the study [21]. The study included 22 patients treated with probiotics and 22 treated with placebos (maltodextrin) [21]. The participants were assessed at weeks six and 10. The subjects were asked not to change their eating habits, and their exercise and physical activity levels were not measured. Participants’ baseline intestinal microbiota compositions were measured at the beginning of the study, with no major differences found between the two groups [21]. At weeks six and 10, 22 patients in the *Bifidobacterium longum* group showed a decrease of over two points in their Hospital Anxiety and Depression (HAD)-depression scores, representing 64% of the group compared to 35% of patients in the placebo group (p = 0.04) [21]. However, there was no significant difference (greater than two points) in HAD-anxiety scores between the two groups [21]. The study results suggest that patients with IBS symptoms had a higher rate of improvement in their depression scores [21]. Moreover, quality of life improved for those in the treatment group compared to those in the placebo group. The group given *Bifidobacterium longum* had reduced amygdala response to fearful faces on brain imaging [21]. There were no differences in inflammatory cytokines (CRP, tumor necrosis factor-alpha (TNF-alpha), IL-6, or brain-derived neurotropic factors). A limitation of the study was that the baseline depression scores were different between the groups, with lower scores being found in the placebo group even though a statistically significant result favored the treatment group when adjusting for these differences [21].

A randomized triple-blind (statisticians, volunteers, and investigators) clinical trial was conducted on adult patients with IBS to study the effect of probiotics with a daily capsule containing a high (1010) or low (109) CFU of *Lactobacillus acidophilus* versus a placebo consisting of microcrystalline cellulose [22]. The study measured the effect based on the change in patients’ Irritable Bowel Syndrome Symptom Severity Scores (IBS-SSS) and Hospital Anxiety and Depression Scores (HADS). Patients with severe IBS symptoms, those who were on laxatives, antibiotics, or anticholinergics, and those who used drugs or alcohol, were pregnant or breastfeeding, or had organic gastrointestinal disease were excluded from the study. A total of 340 subjects completed the trial over 12 weeks of treatment and a four-week washout period of no treatment [22]. Treatment groups had demographic characteristics with a similar baseline, including similar lifestyle habits, diet, exercise, alcohol, and smoking levels. After the treatment period, no significant difference was found from baseline to the end in the treatment group compared to the placebo group [22]. Although the HAD-anxiety score decreased in the treatment group with a mean value of -0.06 change in low dose and -1 change in high dose, no statistical significance level was reached with a p-value > 0.05 for both treatments compared to the placebo group [22]. Similarly, the treatment group experienced a decrease of -0.3 in HADS-depression score for the low dose *Lactobacillus acidophilus* and -0.4 for the high dose [22]. However, no significance was achieved with a p-value > 0.05 for both treatments compared to the placebo [22]. These results may be due to the baseline characteristics of patients with low HAD scores of less than 7 in both depression and anxiety, which does not meet the generalized indication of even mild anxiety or depression [22].

The effect of a psychobiotic on body composition and anxiety in adult patients was studied in a randomized clinical trial using the impedance index (cm2/resistance) and Hamilton Anxiety Rating Scale [23]. The study recruited 45 subjects who were randomly divided into three groups: psychobiotic treatment, dietary treatment, which followed a hypocaloric diet, and a combined treatment group of both psychobiotic and dietary groups. The subjects were followed for three weeks and assessed while maintaining their usual lifestyle habits.

Three grams of psychobiotics containing several bacteria, including 1.5 × 1010 CFU of *Streptococcus thermophilus*, 1.5 × 1010 CFU of *Lactobacillus bulgaricus*, 1.5 × 1010 CFU of *Lactococcus lactis*, 1.5 × 1010 CFU of *Lactobacillus acidophilus*, 1.5 × 1010 CFU of *Streptococcus thermophiles*, 1.5 × 1010 CFU of *Lactobacillus plantarum*, 1.5 × 1010 CFU of *Bifidobacterium lactis*, 1.5 × 1010 CFU of *Lactobacillus reuteri*, maltodextrin from corn, anticaking agent (silica), casein, lactose, and gluten, were taken orally one time per day [23]. Exclusion criteria included pregnancy, diabetes, antibiotics, smoking, and drug or alcohol abuse. No significant difference was observed at baseline between subjects [23]. After 15 patients dropped out of the study, 30 patients completed the study and were distributed randomly into the psychobiotic group, dietary group, or combined treatment [23]. Eleven participants were included in the psychobiotic group, 10 were included in the dietary group, and nine were included in the combined treatment [23]. The study found a significant decrease in impedance index in the psychobiotic group 51.52 - 50.53 (p = 0. 03), a decrease of 55.11 - 51.93 in the combined group (p = 0.01,) and no significant change in the dietary group [23]. A decrease in the Hamilton Rating Scale (HAM-A) from 11 to 7 (p = 0.01) was found in the psychobiotic group, while the combined group decreased from 6 to 5 (p = 0.04) [23]. No significant change was found in the HAM-A score for the dietary group [23].

A randomized, double-blind clinical trial was conducted to study the effect of probiotics on symptoms of depression, metabolic profiles, and serum CRP in patients with major depressive disorder [24]. Patients in the probiotic treatment group took daily capsules containing 2 x 109 CFU of *Lactobacillus acidophilus*, *Lactobacillus casei* (2 x 109 CFU), and *Bifidobacterium bifidum* (2 x 109) for eight weeks. The placebo contained starch [24]. Forty patients aged 20-55 years were recruited for the study, with 20 patients included in each treatment group. Patients were told not to change their diet or physical activity throughout the study. Exclusion criteria included patients who were pregnant, those with a history of coronary infarction or angina, and those with a history of substance abuse. After eight weeks, patients who received probiotics experienced a BDI score decrease of 5.74 (p = 0.001) compared to the placebo [24]. Additionally, serum insulin levels decreased by 2.3 uIU/mL (p = 0.02), and CRP concentrations decreased by 1138.7 ng/mL (p = 0.03) compared to the placebo [24].

The effect of the *Lactobacillus* probiotic on physical stress responses in medical students who were taking a fourth-year authorized nationwide examination was studied in a double-blind, randomized clinical trial [25]. All students had normal baseline HADS-anxiety and HADS-depression scores [25]. Twenty-four students consumed fermented milk with the probiotic one time per day, while 23 students consumed placebo milk. The treatment group was given 100 ml of fermented milk with 1 x 109 *Lactobacillus casei*, while the placebo group was given milk without the probiotic [25]. Exclusion criteria included individuals who were over the age of 30 years, those who smoked, and/or those with a mental health disorder. Patients kept a daily self-evaluation diary for eight weeks. The study found that beginning in weeks five and six, the treatment group reported a significantly lower rate of physical symptoms, with 3/24 patients reporting physical symptoms compared to the placebo group (9/23 patients) (p = <0.05) [25]. Beginning in weeks seven and eight, the probiotic group also reported a significantly lower rate of physical symptoms, with 3/24 reporting physical symptoms compared to 12/23 in the placebo group (p < 0.01) [25]. The study also reported no significant difference in HADS-depression, HADS-anxiety, or salivary cortisol levels during the study period [25]. Interestingly, the logarithmic levels of fecal serotonin were significantly higher in the probiotic group (approximately 3.5 log ng/ml) compared to the placebo group (approximately 3 log ng/ml) two weeks after the examination (p < 0.05) [25].

A case-control study focused on sex differences in microbiota in adult patients with major depressive disorder [26]. Twenty-four female patients diagnosed with major depressive disorder and 24 healthy female patients, along with 20 male patients diagnosed with major depressive disorder and 20 healthy male patients, were used in a comparative study [26]. The patients with major depressive disorder were not treated prior with antidepressant therapy. Fecal samples were collected, and regions of 16S ribosomal ribonucleic acid (rRNA) were extracted, which were then amplified using a PCR using universal primers containing linker sequences. *Actinobacteria* were found to be significantly higher in females with major depressive disorder compared to healthy female subjects [26]. In males with major depressive disorder, *Bacteroidetes* were significantly decreased compared to healthy male subjects [26].

A randomized, double-blind clinical trial was conducted to study the effect of *Lactobacillus rhamnosus* probiotics on depression and anxiety in women during pregnancy and in the post-partum period [27]. Women were randomized to receive the placebo (corn-derived maltodextrin) or probiotic *Lactobacillus rhamnosus* at a dose of 6 x 109 CFUs [27]. The treatments were taken once a day from enrollment at 14 to 16 weeks gestation until birth and from birth until six months post-partum while breastfeeding. Mothers were interviewed at 14 to 16 weeks gestation and once their infants were six and 12 months old. Mothers completed questionnaires about their psychological well-being up until their infant was one to two months old. Depression was measured using the Edinburgh Postnatal Depression Scale, a 10-item screening questionnaire that is used to assess mood. Stress was measured using the State-Trait Anxiety Inventory, a six-item scale. A total of 380 subjects completed the study, 193 (91.0%) in the treatment group and 187 (88.6%) in the placebo group [27]. The study found that women in the probiotic group had significantly lower depression scores (7.7) versus the placebo group (9.0) (p = 0.037) [27]. The probiotic group also had a significantly lower anxiety score (12) compared to the placebo group (13) (p = 0.014) [27]. Additionally, rates of clinically relevant anxiety (score > 15) were found to be significantly lower in the probiotic treatment group versus the placebo group (odds ratio = 0.044; p = 0.002) [27]. The odds ratio is defined as the odds that an outcome will occur given a particular exposure compared to the odds of the outcome occurring in the absence of that exposure [27].

Discussion

Efficacy of Probiotics and Prebiotics on Depression and Anxiety

This literature review suggests that the type of gut microbiota individuals have has a significant effect on their well-being and directly impacts levels of anxiety and depression [22]. Studies have shown that individuals treated with prebiotics and probiotics experience improved physiological outcomes compared to their counterparts [22]. Furthermore, depression and anxiety have a significant emotional and economic impact globally. The role of prebiotics and probiotics should be further explored, especially since it appears that these treatment options have fewer adverse side effects compared to traditional anti-depressants. Of the nine clinical trials that treated patients with probiotics or prebiotics, eight of the studies resulted in significantly improved mood, as evidenced by a decrease in anxiety and depressive symptoms. The study by Lyra et al. (2016) found no significant change in patients’ moods at the start of the study, but these patients did not have a clinical level of anxiety or depression at baseline, which suggests that the results could have been skewed to begin with due to the lack of symptom severity [22].

Overall, the majority of the studies examined suggest strong evidence in support of this paper’s hypothesis. Clinical relevance was shown in the reduction of several scales of severity, including the BDI scale, the Hamilton Rating Scale for Anxiety or Depression, the Edinburgh Postnatal Depression Scale, the State-Trait Anxiety Inventory, and the reported mood of the patients. In addition to mood changes, significant changes in important chemicals were found after treatment with probiotics and prebiotics, including increased fecal serotonin, decreased insulin, a decrease in CRP, and lowered kynurenine-to-tryptophan ratios [17,25]. These results suggest that a positive mood appears to be directly correlated with various chemical changes, for example, a reduction in inflammatory cytokines like CRP or an increase in the availability of tryptophan for serotonin production [17]. A 2009 study by Peluso et al. showed that depression and anxiety have been associated with amygdala hyperactivity [28]. The relationship between the amygdala and the brain appears to be directly impacted by the use of prebiotics and probiotics [28]. Patients who received treatment with prebiotics and probiotics showed a decreased fear response and, as a result, a decreased level of anxiety [28]. Pinto-Sanchez et al.’s study implied that when there are changes in the microflora after the use of prebiotics and probiotics, there is a decrease in catecholamine production, which decreases anxiety by lowering one’s “flight or fight” response [21].

Depletion of serotonin contributes to the pathophysiology of depression, as it is important in mood regulation. Tryptophan is a precursor to serotonin, and a deficiency of tryptophan can lead to serotonin deficiency [29]. The relationship between serotonin and depression has been studied extensively. To determine the effects of tryptophan depletion, a double-blind cross-over study was conducted on 15 women who suffered multiple episodes of depression, had recovered, and were no longer on drug treatment [29]. Patients were given amino acid mixtures that contained a nutritional mixture but lacked tryptophan and were scored on a Hamilton Rating Scale for depression seven hours after drinking each mixture. After treatment with the tryptophan-free mixture, 10 of the 15 women experienced temporary clinically significant depressive symptoms [29]. The mean increase in HAM-D score was 7.3 points higher in the tryptophan-free mixture versus 0.15 for those given the tryptophan mixture (p < 0.001) [29].

It is important to note that the studies varied based on their use of specific prebiotics and/or probiotics and their combination of the two. *Lactobacillus casei* was used in Akkasheh et al.'s and Kato-Kataoka et al.'s studies, and an additional randomized clinical trial by Benton in 2007 found subjects to have improved mood after taking a probiotic with *Lactobacillus casei* [24,25]. *Bifidobacterium longum* was used in more severe cases of clinical depression and was found to improve mood and decrease depressive symptoms. Another randomized clinical trial showed reduced stress-induced symptoms in patients treated with *Bifidobacterium longum* [30].

*Lactobacillus helveticus* and *Bifidobacterium longum* were studied in a double-blind, randomized clinical trial and were found to reduce anxiety-like behavior in rats and decrease psychological distress in humans [31]. Another study designed to test the efficacy of *Lactobacillus helveticus* on rats found that rats treated with *Lactobacillus helveticus* experienced reduced inflammatory markers such as nitric oxide synthase and interleukin-1 [22]. Additionally, the rats had decreased 5-HT metabolism and improved cognitive functions [31]. Although some strains are common in many probiotic treatments, many of these studies use a combination of multiple strains, making it difficult to ascertain the efficacy of each individualized strain.

Additionally, the improvement in symptoms of depression and anxiety after probiotic therapy is likely multifactorial. In addition to increasing monoamine availability, there are other ways probiotics are likely to improve symptoms. Probiotics can reduce the stress response from the hypothalamic-pituitary axis in different ways. As discussed earlier, stress induces intestinal permeability and translocation of lipopolysaccharides, both of which contribute to depressive symptoms. For example, a study found that rats exposed to stress experienced increased adrenocorticotropic hormone, corticotrophin-releasing factor, and lipopolysaccharide (LPS) levels [32]. After treatment with *Lactobacillus farciminis*, there was a suppression in hyperpermeability of the intestines and reduced LPS levels [32]. A reduction in inflammation and inflammatory cytokines results in a reduction of depressive and anxious symptoms. Another study on rats treated with *Bifidobacterium infantis* showed a significant reduction in plasma concentrations of TNF-alpha and IL-6 cytokines and an increase in plasma concentrations of tryptophan [33]. In addition to reducing inflammatory cytokines, which contribute to depression, the increase in serotonin precursors also improves depression symptoms [33].

Altered Composition of Gut Microbiota in Patients With Depression and Anxiety

In addition to probiotic or prebiotic treatment and their role in improving symptoms of mental health disorders, the actual composition of the microbiota in people with these disorders may be altered prior to treatment. In the study done by Chen et al., females who suffered from depression were found to have significantly increased *Actinobacteria* compared to healthy females [26]. Moreover, males who suffered from depression were found to have decreased *Bacteroidetes*. Another study conducted to determine the composition of the gut microbiota in patients with major depression disorder (not gender-specific) found that patients with depression had a relative abundance of *Actinobacteria* with a decrease in *Bacteroidetes* [34].

This indicates that depression may disturb the composition of the gut microbiome. The study also found that when microbiota was taken from patients with major depression disorder and put in germ-free mice, the mice showed depression-like behaviors, demonstrating the relationship between gut composition and its effects on mood [34].

Limitations

Most of the trials and studies had small sample sizes, which decreased the overall power of the study. It would be beneficial to conduct these experiments on a larger population size to increase the clinical significance of the findings. Additionally, many of the assessments relied on subjective recording to come up with measurable impacts from the treatment groups. The period of the studies was not unilateral, with many of the studies having different treatment lengths. It would also be useful to determine how varying lengths of time would change the results.

The strains of prebiotics and probiotics varied widely throughout the studies, with some overlap between bacterial strains treated in more than one study. Some common bacteria that overlapped included *Lactobacillus* and *Bifidobacterium*. Due to the multiple combinations of strains used in studies, it is difficult to ascertain the effect of individual strains. Further studies could be performed to determine the efficacy of individual strains and their impact on mood. Dosing was another independent variable; therefore, it is difficult to determine if the amount of a strain or CFUs had a direct effect on mood.

Additionally, it would be beneficial if there were further studies to determine how confounding factors affect mood. For example, sleep was a measurement that was not included in the studies. Depression and anxiety significantly impact sleep and could have been affected by probiotics or prebiotics, making this an important topic for future studies. The definitions and measurements of depression and anxiety also varied across the studies and included the BDI scale, the Hamilton Rating Scale for Anxiety or Depression, the Edinburgh Postnatal Depression Scale, the State-Trait Anxiety Inventory, and subjective symptoms. The baseline characteristics of the subjects in the studies also varied in significant ways. For example, some studies did not allow participants who had taken antidepressants to participate while others did [21]. It should also be further investigated whether the use of selective serotonin reuptake inhibitor (SSRI) medication can also affect and directly impact gut microflora. Another difference in studies was the patients’ baseline diet, which was not restricted in some studies. It would be worthwhile to explore how different diets affect mood since diet itself can directly affect the gut microflora.

Furthermore, baseline microbiota varied between subjects, and no particular baseline was established. However, the study by Azpiroz et al. tested for major strains and ensured similarity between subjects [20]. This is important to note, since the composition of the gut microbiota has significant implications for mood, as discussed earlier [20]. On the other hand, the study by Azpiroz et al. had patients with no baseline depression, which may have been why there was no improvement in depressive symptoms [20]. However, the subjects did have anxiety, and after treatment with prebiotics, there was a significant improvement in anxiety symptoms [20]. Alternatively, the study by Pinto-Sanchez et al. included patients with depression but not anxiety, and the results showed an improvement in depression [21]. This implies that individuals with higher severity of symptoms were more likely to see improvement [21].

Lastly, the level of physical activity, which also plays a role in gut microbiota and mental health, was not similar between patients. The time of year the studies were conducted was also different, and it is well known that there is a seasonal change in vitamin D levels, which directly impacts mood. These variations could have had an effect on the treatment outcomes. The studies were mostly done on adults, and the effect of prebiotics and probiotics on children or teenagers has not been thoroughly tested.

Future implications

The clinical effects of these treatments need to be studied comprehensively in larger sample sizes over longer periods of time with uniform characteristics for the severity of disease and other baseline characteristics to determine the generalizability of the results. Additionally, standardized dosing and strains should be used. The use of probiotics and prebiotics as supplements to conventional antidepressant medication, especially for treatment-resistant depression and anxiety, should be further investigated. Additionally, more research should be carried out to determine if similar effects would be seen in younger populations (below 18 years). Fecal microbiota transplants from healthy patients to patients with depression or anxiety may also prove to be efficacious. Lastly, it should be further explored how strains impact patients’ moods when they have both anxiety and depression.

## Conclusions

The evidence shown in this review suggests that, when treated with prebiotics and probiotics, individuals with anxiety or depression can improve their mood and decrease the severity of their symptoms. This is done by multiple mechanisms, but largely through attenuating the inflammatory response and increasing serotonin availability. The results of these studies suggest that prebiotics and probiotics may have a role to play in the treatment of these mental illnesses.

## Appendices

**Table 1:** Evidence table for literature review

Author	Date of publication	Study design	Level of evidence		Study population	Objective	Results	
Kazemi et al. [17]	2018	Randomized clinical trial	1	110 subjects		Compare the effects of supplementation with prebiotic and prebiotic on the Beck’s Depression Inventory (BDI) score as a primary outcome to a placebo group	Eight weeks of probiotic supplements with depression disorder had a decrease in BDI score of 18.25 baseline - 9.0 end compared to placebo of 18.74 - 15.55, and prebiotic 19.43 baseline - 14.14 end	
Azpiroz et al. [20]	2017	Randomized clinical trial	1	79 subjects		Determine the effects of supplementation with short-chain fructooligosaccharide (scFOS) compared to placebo on anxiety in patients with irritable bowel syndrome	Patients treated with scFOS had significantly reduced anxiety scores	
Slykerman et al. [27]	2017	Randomized clinical trial	1	380 subjects		Evaluate the effect of *Lactobacillus rhamnosus* given during pregnancy and postpartum on symptoms of maternal depression and anxiety postpartum and compare it to a placebo	Patients treated with the probiotic reported lower depression scores (7.7) compared to the placebo (9), as well as lower anxiety scores (12) compared to the placebo (13)	
Ghorbani et al. [19]	2018	Randomized clinical trial	1	40 subjects		Assess the change in Hamilton Rating Scale for Depression after synbiotic supplementation to fluoxetine	Greater reduction in Hamilton Rating Scale in patients treated with synbiotic supplementations (-19.25) compared to only taking fluoxetine (-17.75) after six weeks	
Colica et al. [23]	2017	Randomized clinical trial	1	45 subjects		Evaluate the effects of psychobiotics on anxiety	The Hamilton anxiety rating scale showed a significant reduction after treatment in the study population	
Kato-Kataoka et al. [25]	2016	Randomized clinical trial	1	47 subjects		Investigate the effects of the probiotic *Lactobacillus casei* strain on stress in medical students taking a nationwide examination	Higher level of fecal serotonin levels in the treatment group as well as a reduction in physical symptoms of abdominal discomfort	
Lyra et al. [22]	2016	Randomized clinical trial	1	340 subjects		Evaluate the effects of *Lactobacillus acidophilus* on irritable bowel syndrome and quality of life related to anxiety and depression	Irritable Bowel Syndrome Symptom Severity Score decreased in all patients in the treatment group	
Chen et al. [26]	2018	Case-control	3	88 subjects		Investigate whether there were sex differences in gut microbiota in patients with major depressive disorder (MDD)	Results showed there were significant sex differences in gut microbiota in patients with MDD	
Pinto-Sanchez et al. [21]	2017	Randomized clinical trial	1	44 subjects		Evaluate the effects of *Bifidobacterium longum* on anxiety and depression in patients with irritable bowel syndrome	The treatment group had a reduction in depression scores on the Hospital Anxiety and Depression scale	
Akkasheh et al. [24]	2016	Randomized clinical trial	1	40 subjects		Compare the effects of probiotic intake on symptoms of depression in patients with major depressive disorder	Patients who received probiotic supplements had significantly decreased Beck’s Depression Inventory scores compared to placebo	
